# A Modified Tension Band Fixation Technique for the Management of Patellar Fractures Using Crossed Pins and a Lateral Parapatellar Approach

**DOI:** 10.7759/cureus.24546

**Published:** 2022-04-28

**Authors:** Freideriki Poutoglidou, Matija Krkovic

**Affiliations:** 1 Addenbrookes Major Trauma Unit, Department of Trauma and Orthopaedics, Cambridge University Hospitals NHS Trust, Cambridge, GBR

**Keywords:** lateral approach, crossed pins, tension band, fractures, patella

## Abstract

The use of the tension band technique for patellar fracture fixation has been associated with a loss of the rigidity of the construct after cyclic loading. Biomechanical studies have shown the biomechanical superiority of the crossed pin configuration relative to the traditional parallel one. Here, we describe a modified tension band technique that involves the use of crossed pins and a figure-of-eight passed as close to the bone as possible through a lateral parapatellar approach. The basic surgical technique and our case series are reviewed.

## Introduction

The tension band technique relies on the principle that applying an implant, such as a wire or a plate, on the tension side of the bone results in the conversion of the tensile forces into compression forces, provided that the compression cortex is sufficient [1]. Tension band wiring for patellar fractures has been associated with a loss of the rigidity of the construct after cyclic loading [2-4].

The patellofemoral joint is a highly complex structure. First, the contact area between the patella and the femoral condyles increases and moves proximally during flexion [5]. Second, the patellofemoral joint reaction force is variable and depends on several factors, including the knee joint angle, the muscle tension, and the distance between the center of gravity and the joint [6]. Finally, patellar tracking is a very complicated motion that involves shift, rotation, and tilt [7]. The patella slides on a convex surface [8], which results in an uneven distribution of the forces in its anterior and posterior cortex. Although a parallel k-wire configuration may achieve adequate compression on the posterior surface of the patella, some displacement will occur on the anterior surface. A recent biomechanical study has shown that a crossed k-wire configuration provides greater fixation stiffness under cyclic loading and improves interfragmentary compression compared to the traditional parallel one [9].

Here, we describe a modified tension band technique for the management of patellar fractures. The technique involves a crossed pin configuration and a lateral parapatellar approach.

## Technical report

Surgical technique

**Figure 1:**
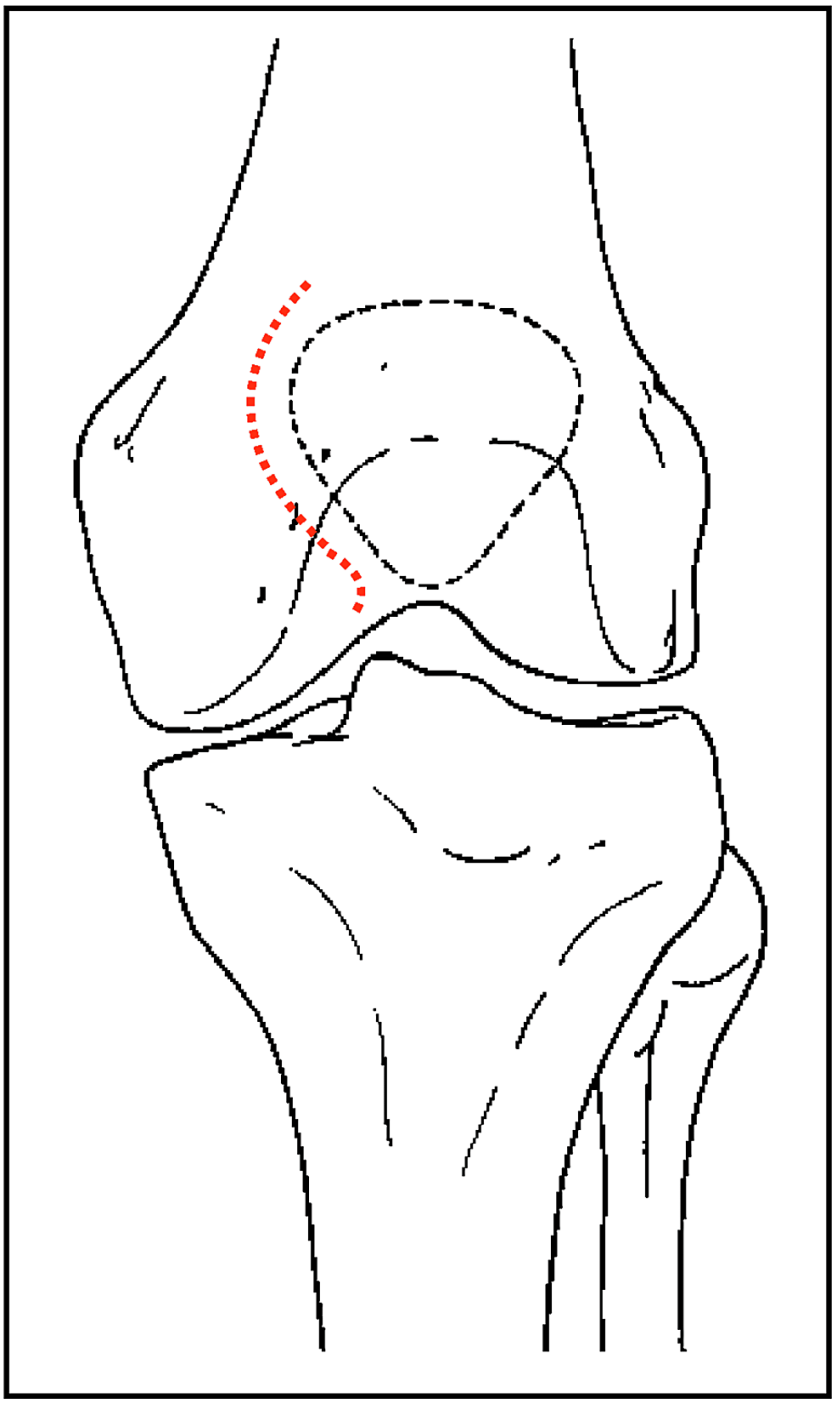
Incision (red dotted line) for the modified crossed pin tension band technique.

**Figure 2:**
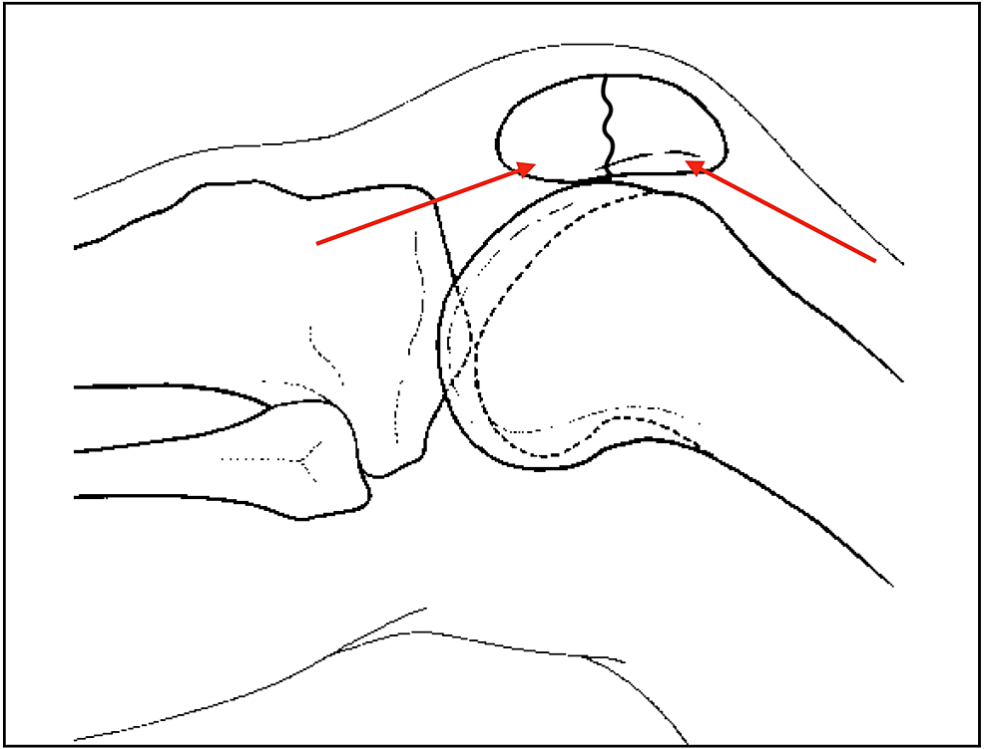
Schematic description of the entry points of the k-wires. K-wires are inserted for the proximal fragment from proximal and lateral and exit in the centre of the fragment and for distal fragment from distal and lateral to the centre of the fragment. Both wires are aimed close and parallel to the subchondral bone. Red arrows: direction of the pins.

Preoperative anteroposterior and lateral radiographs are obtained, as well as a CT scan if needed. The procedure is performed under fluoroscopy. The patient is placed in a supine position and a tourniquet is applied to the proximal thigh (surgeon’s preference). A lateral parapatellar approach is performed, in particular, an anterolateral approach is made (Figure 1), followed by lateral parapatellar arthrotomy and exposure of the fracture margins. Next, two 1.6-2.0 mm k-wires are inserted for the proximal fragment from proximal and lateral and exit in the centre of the fragment, and for the distal fragment from distal and lateral to the centre of the fragment. Both wires are aimed close and parallel to the cartilage/subchondral bone for both fragments (Figure 2). At this stage, fragments are reduced and retained in place with a pointed reduction forceps. The k-wires are then progressed into the opposite fragments with a power tool until they both exit the opposite fragment (1-2 cm max) on the medial side. The wires will exit into the quadriceps/patellar tendon and will not be directly visible. The reduction is verified with palpation of the retropatellar surface through the lateral parapatellar approach. An image intensifier (direct, lateral, and oblique views) is used to verify the reduction and the appropriate placement of the k-wires. Care is taken to avoid k-wires crossing at the level of the fracture, as this may compromise rotational stability. Whether wires are bracing the joint/articular cartilage is decided based on direct examination and not on an image intensifier.

Small vertical incisions are made through the quadriceps and patellar tendons on the medial side to identify the k-wires. When identified, the k-wires are backed out to allow just enough length to allow the passage of a secure tension band wire. Tension band wire is then placed in the standard way but has to go through the tendons to minimize the potential of the tension band wire cutting through the tendon on tensioning. The author’s preferred method is to put the wire first on the proximal fragment from medial to lateral, followed by the distal fragment from lateral to medial. Appropriate management of the tension band wire is required to prevent unnecessary repetitions of the task. Tension band wire is tensioned using a standard tensioning method. When it is confirmed that the tension wire is in the appropriate place behind both k-wires on the medial side of the patella, both k-wires are bent, rotated into the correct position, and backed out to get the loop of the k-wires as close to the patella as possible. The same is repeated on the lateral side, with the main caveat that the k-wires should be cut at the appropriate length and bent directly into the most appropriate position, and that twisting of the wires will affect the position of loops on the medial side. Incisions in the tendons are closed with a reabsorable suture. The final reduction is confirmed by palpating the joint from inside through the lateral arthrotomy if the joint laxity allows. Finally, the position of the k-wires and the figure-of-8 are checked with the image intensified and the wound is closed in layers.

Full weight-bearing is typically initiated immediately post-surgery. Patients have a hinged knee brace locked in extension for two weeks. The brace is opened in progressively increasing flexion two weeks post-surgery.

Case series

**Figure 3:**
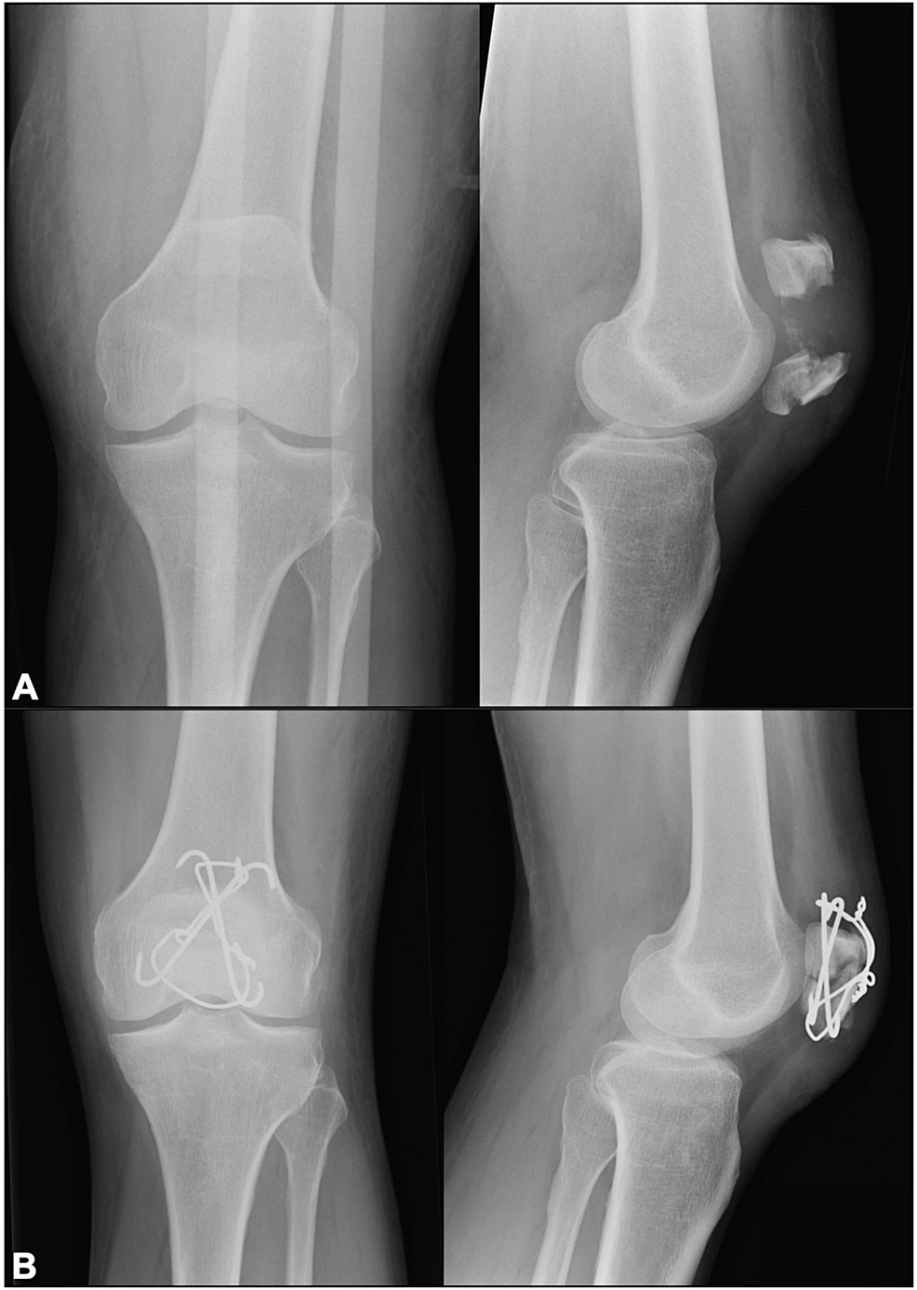
Preoperative (A) and postoperative (B) anteroposterior and lateral X-rays of a patellar fracture managed with the modified crossed pin tension band technique.

**Figure 4:**
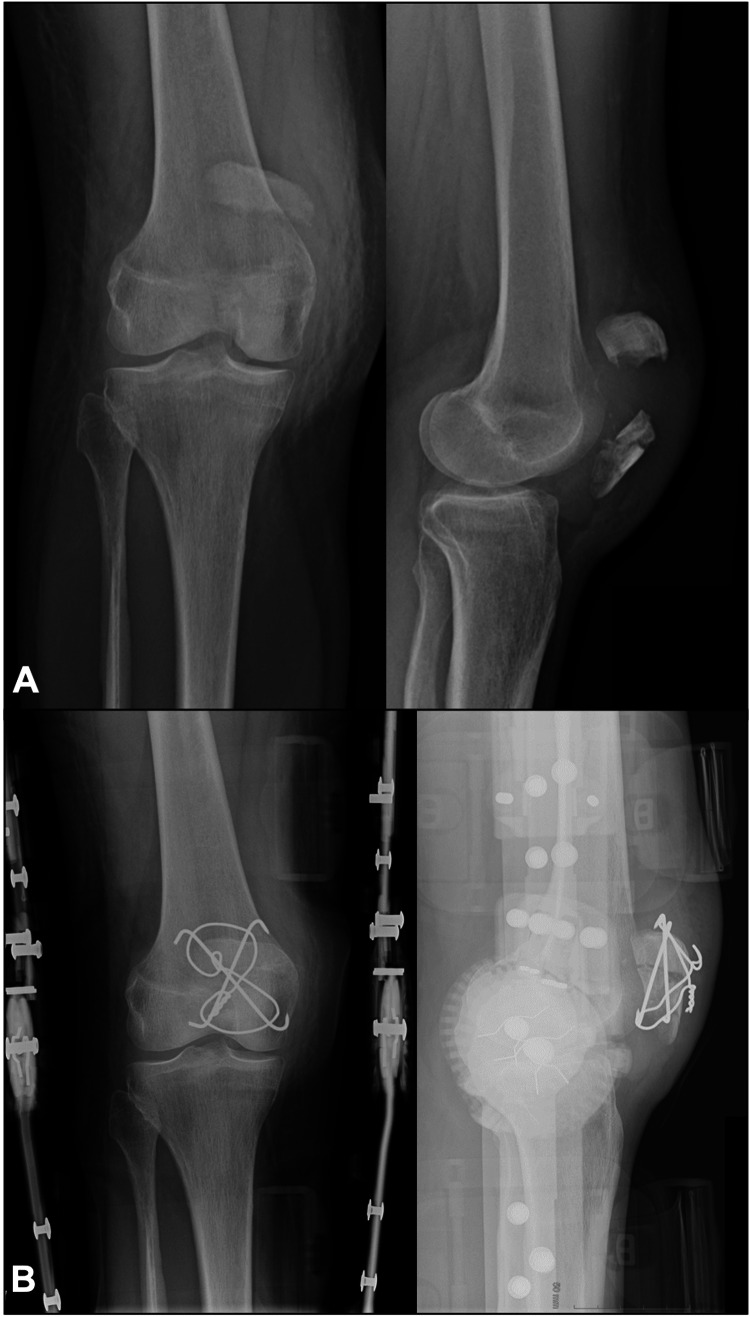
Preoperative (A) and postoperative (B) anteroposterior and lateral X-rays of a comminuted patellar fracture treated with the crossed pin tension band technique.

**Figure 5:**
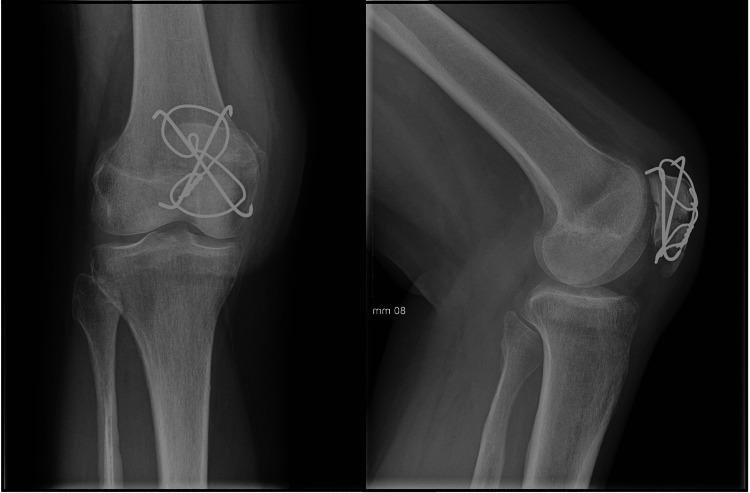
Anteroposterior and lateral X-rays of a healed patellar fractures four months after fixation with the modified crossed pin tension band technique.

Six patients with patellar fractures were treated operatively with this technique. Four of the patients were male, and two of them were female. The mean age of the patients was 41.17 years (a range of 20-60). Five of the patients had comminuted fractures, and one of them had a transverse fracture pattern. There were no intraoperative complications. The average follow-up of the patients was 7.83 months (range 2-22). Radiographic union was achieved in all patients, and there were no infections. None of the patients complained of stiffness, anterior knee pain, or symptomatic hardware. One of the patients had an implant removed. This patient, however, had concomitant lower limb fractures and multiple operations in the following months. Representative X-rays of patients treated with the crossed pins tension band technique are shown in Figures 3-4. Figure 5 demonstrates a healed patellar fracture four months postoperatively.

## Discussion

The tension band technique has been associated with high complication rates and an early mobilization may require a more rigid construct. Several studies have shown a displacement in the fracture site after cyclic loading [2,3], and the reason for this may be the fact that forces act on different areas of the patella through knee flexion. Patella tracking occurs on a convex surface, and fracture displacement on the anterior surface of the patella is inevitable. Crossed pins have been mainly used in the management of phalangeal and paediatric supracondylar humeral fractures and, in both cases, they have been shown to increase stability. Accordingly, a crossed pin configuration may confer a more rigid construct in patella fractures that could resist fragment displacement during flexion. Indeed, Lenihan et al. demonstrated a significantly smaller fracture gap displacement with the use of crossed pins compared to conventional parallel ones [9]. It should be noted that the crossed pin technique does not contradict the AO principles since, according to AO, the resistance to displacement comes from the cerclage wire and not from the pins that act mainly as anchoring points.

A biomechanical study by Baran et al. showed that for optimal stabilization, the tension force transfer should be done directly on the bone and that interposed tendinous tissue reduces stability [10]. Accordingly, in this modified technique, the figure-of-8 wire passes as close to the bone as possible. This way, we increased the stability of the construct and, at the same time, reduced soft tissue trauma. Regarding the approach used, the traditional mid-axial longitudinal approach does not allow palpation of the retropatellar surface. On the other hand, a lateral parapatellar approach not only allows palpation of the articular surface but at the same time decreases the risk of infection since, with this approach, the hardware is not directly under the incision, the incision is smaller, and it involves less soft tissue trauma.

## Conclusions

In conclusion, the tension band technique for the management of patella fractures has been associated with many complications. In this study, we present a modified tension band method with a crossed pin configuration and through a lateral parapatellar approach. Although only a limited number of patients were treated with this technique, the short-term clinical and radiological outcomes are very promising. The crossed pin tension band technique follows sound biomechanical principles, is reproducible, and yields satisfactory outcomes.
